# Dyeing of cotton fabric materials with biogenic gold nanoparticles

**DOI:** 10.1038/s41598-021-92662-6

**Published:** 2021-06-24

**Authors:** M. Sivakavinesan, M. Vanaja, G. Annadurai

**Affiliations:** Environmental Nanotechnology Division, Sri Paramakalyani Centre of Excellence in Environmental Sciences, Manonmaniam Sundaranar University, Alwarkurichi, 627412 India

**Keywords:** Environmental sciences, Natural hazards, Materials science

## Abstract

The present work aimed at synthesizing gold nanoparticles in a biological method employing fruit peel waste dumped in the environment. The peels of *Garcinia mangostana* (Mangostan), were collected from the nearby tourist spot during the season. The collected fruit peels were washed, dried, powder and extracted by using boiling water and acetone. The precipitated extract was dried and powdered for further use. The dried and powdered peel extract was added to the gold solution and boiled to 80 °C and the color change is observed. The color change indicates the completion of the synthesis of gold nanoparticles. The effect of pH, gold ion concentration, peel extract powder concentration, and the temperature was tested by varying the parameters. The biosynthesized nanoparticles were characterized using the UV–Vis spectrophotometer to identify the surface plasmon resonance peaks corresponding to gold nanoparticles. The bio-moieties responsible for the synthesis of gold nanoparticles were identified using the Fourier Transform Infra-Red Spectroscopy. The crystalline nature was detected by using an X-Ray Diffractometer. Atomic Force Microscope viewed the 3D surface image of the gold nanoparticle. The shape and morphology of the nanoparticle were identified by using a Field Emission Scanning Electron Microscope. The active compounds for gold nanoparticle synthesis were identified using Gas Chromatography-Mass Spectrometry. The gold nanoparticle was synthesized in various colors and used for dyeing cotton fabrics. The dyed cotton materials were exposed to various stress conditions to determine the color fastening.

## Introduction

Mangostan (*Garcinia mangostana* Linn.) (Clusiaceae) is a tropical tree distributed in India, Myanmar, Malaysia, Philippines, Sri Lanka, and Thailand. *G. mangostana* grows up to 6–25 m^1^. The fruits of *G. mangostana* show good taste hence it is called as “queen of fruits” and it contains a rich source of phenolic compounds such as xanthones, condensed tannins, and anthocyanins^2,3^. In Ayurvedic medicine, the pericarp of mangostan-fruit has been widely used against inflammation, diarrhea, cholera, and dysentery^4,5^.

Nanotechnology is an extremely powerful emerging technology that is expected to have a substantial impact on technology now and in the future^6^. Before 100 years, Michael Faraday reported the colloidal form of gold nanoparticles. Gold nanoparticles are one of the most extensively studied nanomaterials^7^ and were synthesized through several biomaterials namely *Volvariella volvacea *(Singer)^8^, eggshell membrane^9^, *Stoechospermum marginatum *(Agardh)^10^, *Solanum nigrum *L^11^, *Trichoderma harzianum *(Rifai)^12^, *Brassica oleracea *L.^13^, *Hibiscus sabdariffa *L.^14^, *Couroupita guianensis *(Aubl) fruit extract^15^, *Elaeis guineensis* (Jacq)^16^ and Crocin^17^. The analysis of surface plasmon resonance absorption band can also provide valuable information on the size, structure, and aggregation properties of gold nanoparticles. The dielectric constant of the surrounding medium determines the shape and particle size of the nanoparticle^18^. Gold nanoparticles are employed for various applications including functionalization of bamboo pulp^19^, sensors^20,21^, digital decoration^22^, antioxidants^15^, anti-cancer^17^, catalytic degradation of dyes^23,24^, and colourants^18^.

The dyes disposed of in textiles, cosmetics, food, leather, and plastic industries cause major environmental pollutants which disrupt and cause various toxic effects like mutagenic or carcinogenic in aquatic organisms. The dyes can also accumulate in soil and it takes a higher half-life time for degradation^23^. These negative impacts on the environment make an alarm to research communities and find an alternate solution for dyeing. Moreover, textile products with enriched functionalities like antibacterial, antistatic, stain-resistant, and UV protection are being demanded by consumers^25^.

The surface plasmon resonance property of nano solutions (Gold, silver, and copper) was used for coloring purposes^22^. Metal nanoparticles represent noble bright colors due to the localized surface plasmon resonance. The different colors of noble metal nanoparticles have been used to dye cotton and wool^26^. Nanocrystalline cobalt aluminate spinel was considered as a nano pigment that contains blue color enhanced by the addition of starch solution^27^. Afshari et al.^28^ reported that dyeing of polyester fabric using copper nanoparticles and assessed its antimicrobial property. In the present study, gold nanoparticles were synthesized through a greener method and it was applied for the dyeing process.

## Materials and methods

### Preparation of fruit peel extract powder

The fruit peels of *G. mangostana* were collected from Courtallam (8.9342° N 77.2778° E), Tirunelveli, Tamilnadu, India. Collected fruit peels were washed with distilled water and dried in shade to remove adhering dirt and dust. The fruit peels (100 g) were boiled in distilled water at 90 °C for 30 min and crushed by adding 100 ml of distilled water and the resultant extract was filtered through a clean muslin cloth. Equal volumes of chilled acetone were added to filter the precipitate. The resulting precipitate was collected by centrifugation (Eltek RC4100F) at 7000 rpm for 10 min, air-dried in a hot air oven, powdered form, and used for further experiments.

### Green synthesis of gold nanoparticles

Chloroauric acid (HAuCl_4_) in double-distilled water (1 mM) was used as a source of gold nanoparticles throughout the experiments. The reaction mixture contains 10 mg fruit peel extract powder, 2 ml chloroauric acid solution (1 mM). The reaction mixtures were heated at 80 °C in a water bath for 3 min. These were monitored for different time intervals and the nanoparticles and microstructures formed were characterized further.

### Synthesis of different colours of gold nanoparticle solution

Gold nanoparticles were synthesized in different colors for dyeing applications. The color of the solution depends on the surface plasmon resonance effect (SPR) of the gold nanoparticle synthesized. The different colors of solution were prepared by increasing the concentration of chloroauric acid solution from 10^–2^, 10^–3^, 10^–4^, 10^–5^, and 10^–6^ M.

### Characterization of nanoparticles

The reduction of gold ions was monitored by using a double beam UV–vis spectrophotometer (Lambda 25, Perkin Elmer, Singapore) of the reaction medium in the wavelength range of 450–700 nm with a 10 mm quartz cell. FTIR confirmed functional biomolecules associated with the reduction of gold ions into gold nanoparticles (Spectrum two, Perkin Elmer, Singapore). Biosynthesized gold nanoparticles were centrifuged at 7000 rpm for 15 min and the pellets were washed with distilled water. The centrifuging and re-dispersing process were repeated thrice. The samples were dried and analyzed at a wave region of 400–4000 cm^-1^.

The purified gold nanoparticle structures and compositions were analyzed by XRD (Panalytical X ‘Pert Powder X’ Celerator Diffractometer). The crystalline nature of the nanoparticles was analyzed at the 2θ ranges of 10–80°. The morphology and size of the gold nanoparticles were found by Field Emission Scanning Electron Microscope (Philip model CM 200). Atomic Force Microscopy analysis (Nanosurf EasyScan 2) was done to examine the shape and size of the nanoparticle synthesized using the biological technique.

The mangostan peel extract was subjected to Gas Chromatography-Mass Spectrometry (GC–MS) (Perkin Elmer, Clarus 680) analysis to identify the compounds responsible for the synthesis of gold nanoparticles. The acquisition parameters for GC–MS are, Oven: Initial temp 60 °C for 2.80 min, ramp 10 °C/min to 300 °C, hold 6 min, InjA auto = 260 °C, Volume = 1 μL, Split = 10:1, Carrier Gas = He, Solvent Delay = 2.80 min, Transfer Temp = 230 °C, Source Temp = 230 °C, Scan: 40 to 600 Da, Column 30 × 250 μm. Further, the gold nanoparticles coated cotton fabrics were analyzed for the presence of nanoparticles using a Scanning Electron Microscope (Carl Zeiss, Model EVO 18).

### Gold nanoparticle as dye

#### Thermofixing method

The different colored gold nanoparticles were fixed on the wicks (cotton with length 10 cm) material by thermo fixing method. The wicks pieces were dyed by boiling with gold nanoparticle solution at a higher temperature range from 120 to 160 °C. Initially, the dyed wicks were kept at 120 °C for 15 min without alteration in temperature. After 15 min, the temperature of boiling was increased to 160 °C and kept for 1 h. Fabric materials were rinsed with water and then dried Material was again rinsed well after reduction cleaning and then dried in the air^29^.

#### Colour fastness of the fabrics

The Thermo fixed wick pieces were tested for colorfastness against drastic conditions. Each dyed fabric piece was dried at 45 °C for 24 h and tested for colorfastness against different situations. The color fastness of the colored fabrics was verified by dipping the fabrics in commercial detergent solutions for 24 h and by scouring. Following this process, the pieces were also treated with acid (1% acetic acid), alkali (1% KOH), and hot water with commercial detergent (pH ~ 11) for 1 h. The color fastnesses of the fabrics were also determined by the exposure to sunlight during summer (April–May; average atmospheric temperature 36 °C) at different time scales, i.e. 1–10 days.

## Results and discussion

### Green synthesis and characterization of gold nanoparticles

#### Visual observation

**Figure 1:**
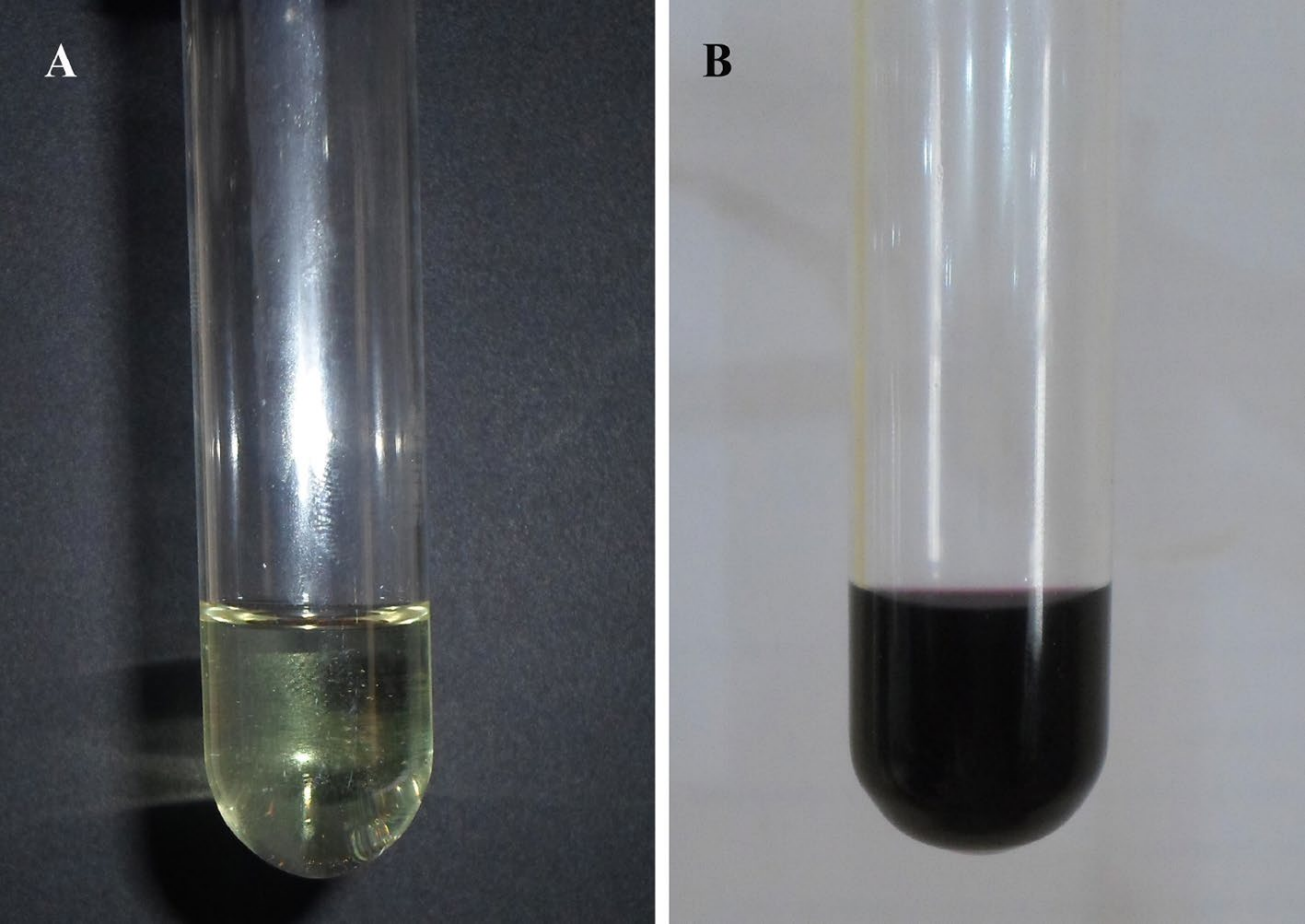
Gold solution (**A**) and gold nanoparticle (**B**) colloidal solution.

Gold nanoparticle formation was primarily identified by color change visually. The observation of color changes, from colorless solution into purple or pink color, was one of the processes to identify the synthesis of gold nanoparticles in the solution (Fig. 1). The peel powder extract was exposed to gold chloride solution and boiled, the green synthesis of nanoparticles started within few minutes and was identified by color change. Gold nanoparticles show different colors for each concentration of the chosen chloroauric acid solution. The color ranges from yellow, purple, and pink. The color arises because of the excitation of the surface plasmon resonance effect of gold nanoparticles. Previously, the report of Singaravelu et al.^30^ and Inbakandan et al.^31^ has also shown that the gold nanoparticle synthesis by using marine algae *Sargassum wightii* and the marine sponge *Acanthella elongate*, respectively. The result color change got in this investigation is very interesting in terms of the identification of potential plants for synthesizing the nanoparticles^32^.

#### UV–vis spectrophotometer analysis (Effect of gold ion concentrations)

**Figure 2:**
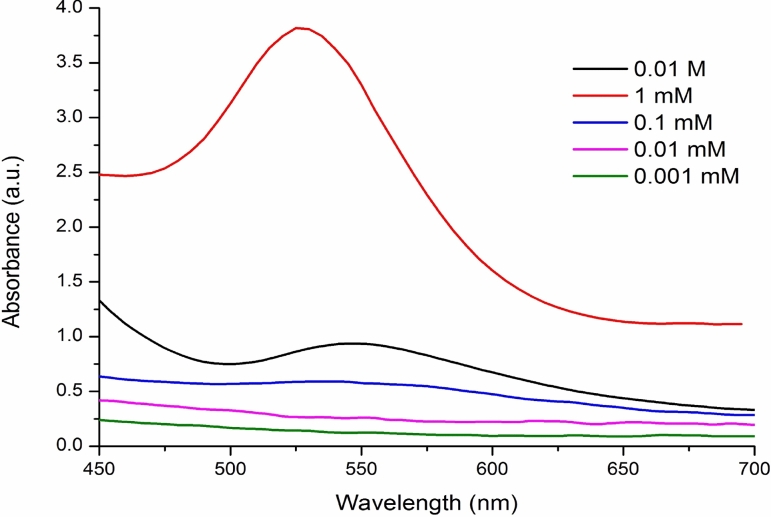
UV–Vis spectroscopy image of gold nanoparticle at various concentrations.

UV–vis spectroscopy is an important preliminary technique used to determine the formation of nanoparticles at different concentrations of gold ions. Figure 2 shows the effect of gold ion concentration in the gold nanoparticles synthesis process by using the peel powder of mangostan. Characteristic surface plasmon absorption bands are observed at 530 nm for the purple-colored gold nanoparticles synthesized from 1 mM (10^-3^ M) gold ion concentration. The sharp peak at 530 nm is the characteristic peak of monodispersed spherical nanoparticles. The 1 mM concentration shows a narrow band with increased absorbance, whereas other concentrations like 10^-4^ M show a broad peak at 547 nm with low absorbance. It reveals large-sized nanoparticles because of the higher multipole plasmon excitation^33,34^. Single SPR band formation with increased absorbance at lower wavelength reveals the synthesis of the small spherical shape of nanoparticles^35^.

#### X-ray diffraction analysis

**Figure 3:**
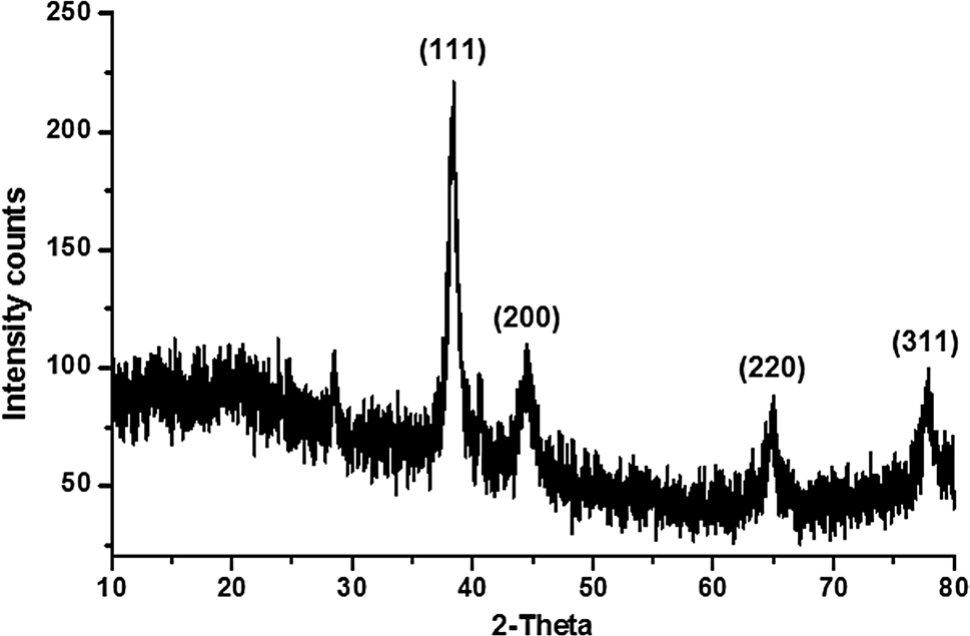
XRD pattern of gold nanoparticle.

The crystalline size and structure of the gold nanoparticles were determined by XRD in the entire spectrum of 2θ values ranging from 10 to 80°. Peel powder extract mediated synthesized gold nanoparticles using mangostan shows four strong diffraction peaks at the 2θ values of 38.30°, 44.51°, 64.82° and 78.56° (Fig. 3) could be assigned the plane of (1 1 1), (2 0 0), (2 2 0) and (3 1 1), respectively shows the gold nanoparticles are face-centered cubic crystalline structure. The synthesized gold nanoparticles are compared with standard gold and pure nanogold particles which are published by Joint Committee on Powder Diffraction Standards (File nos. 04–0783 and 84–0713). A comparison of our XRD spectrum with the standard confirmed that the gold nanoparticles formed in our experiments were nanocrystals. The mean size of synthesized gold nanoparticles was calculated using Debye–Scherrer’s Eq.^36^ by determining the width of the (1 1 1) Bragg reflection using the following equation.

1 $$ {\text{D}} = {\text{K}}{\uplambda} /{\upbeta} \cos {\uptheta} $$

where D is the average crystallite domain size perpendicular to the reflecting planes, λ is the X-ray wavelength (1.5418 Å), β is the width of the XRD peak at half-height, θ is the diffraction angle, and K is the Scherrer coefficient (0.89) shape factor for spherical particles. The calculated average particle size of the synthesized nano gold using mangostan peel powder extract was found to be 128 nm. The present report is well-matched with the standard gold and the report of Singaravelu et al.^30^ and Shukla et al.^37^.

#### Field emission scanning electron microscope

**Figure 4:**
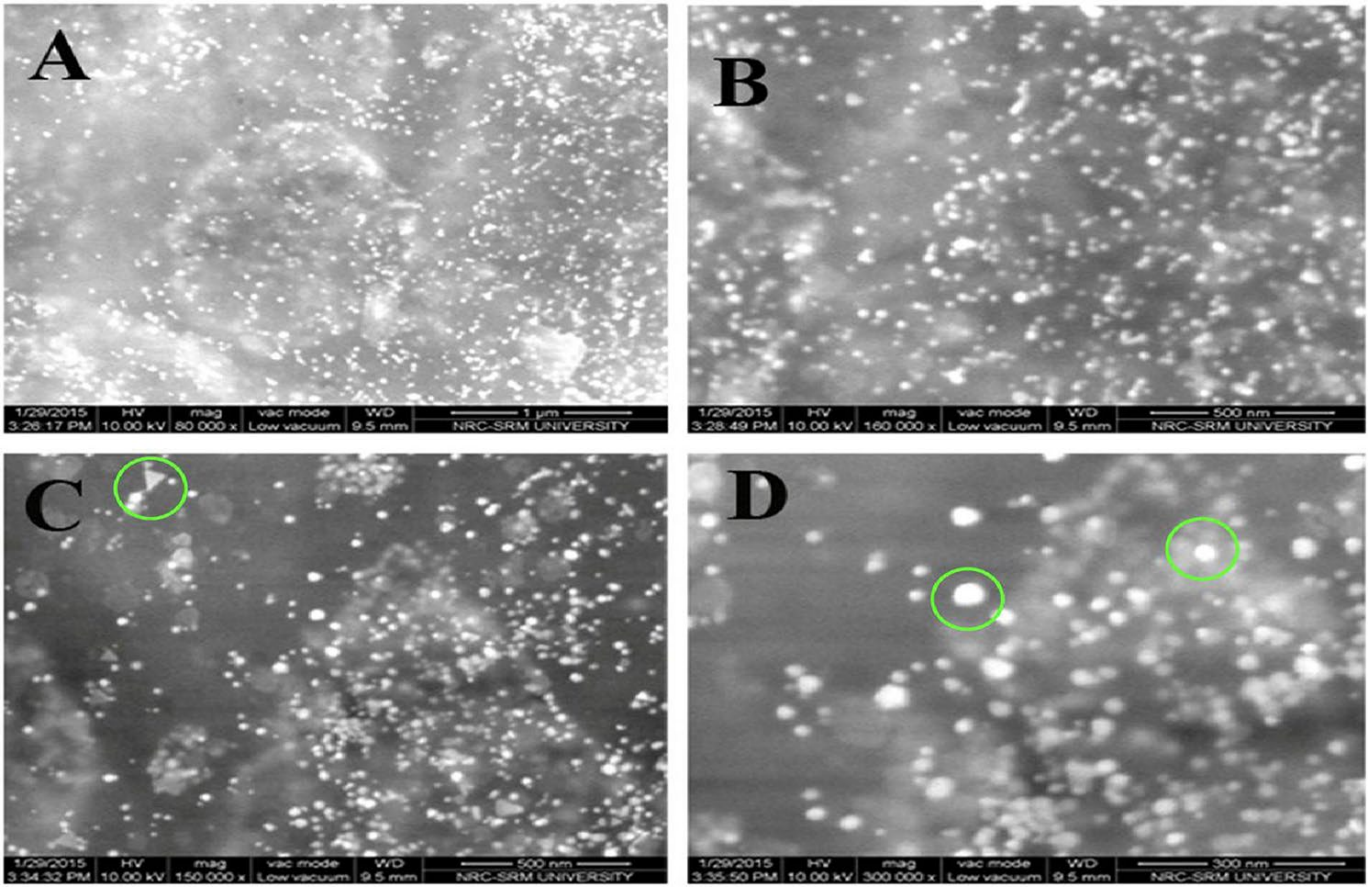
FESEM image of gold nanoparticles synthesized using mangostan peel extract (**A**) 1 µm, (**B**) 500 nm, (**C**) 500 nm, (**D**) 300 nm.

The surface morphology of the synthesized gold nanoparticles was identified on the nanoscale bar by Field Emission Scanning Electron Microscope. FESEM image had shown the shape and distribution of the gold nanoparticles synthesized by using peel extract of mangostan (Fig. 4). FESEM micrographs show synthesized nanoparticles were adsorbed on the surface of peel powder. FESEM image shows individually dispersed gold nanoparticles and several aggregates synthesized with irregular shapes using peel powder extract of mangostan. It illustrates the particles are predominantly spherical and some particles aggregate into larger particles with no well-defined morphology and the size of the gold nanoparticles ranging from 75 to 130 nm. The similar irregular shape of gold nanoparticles with different sizes from 5 to 100 nm was reported by using macerated clove bud's solution^38^.

#### Atomic force microscope

**Figure 5:**
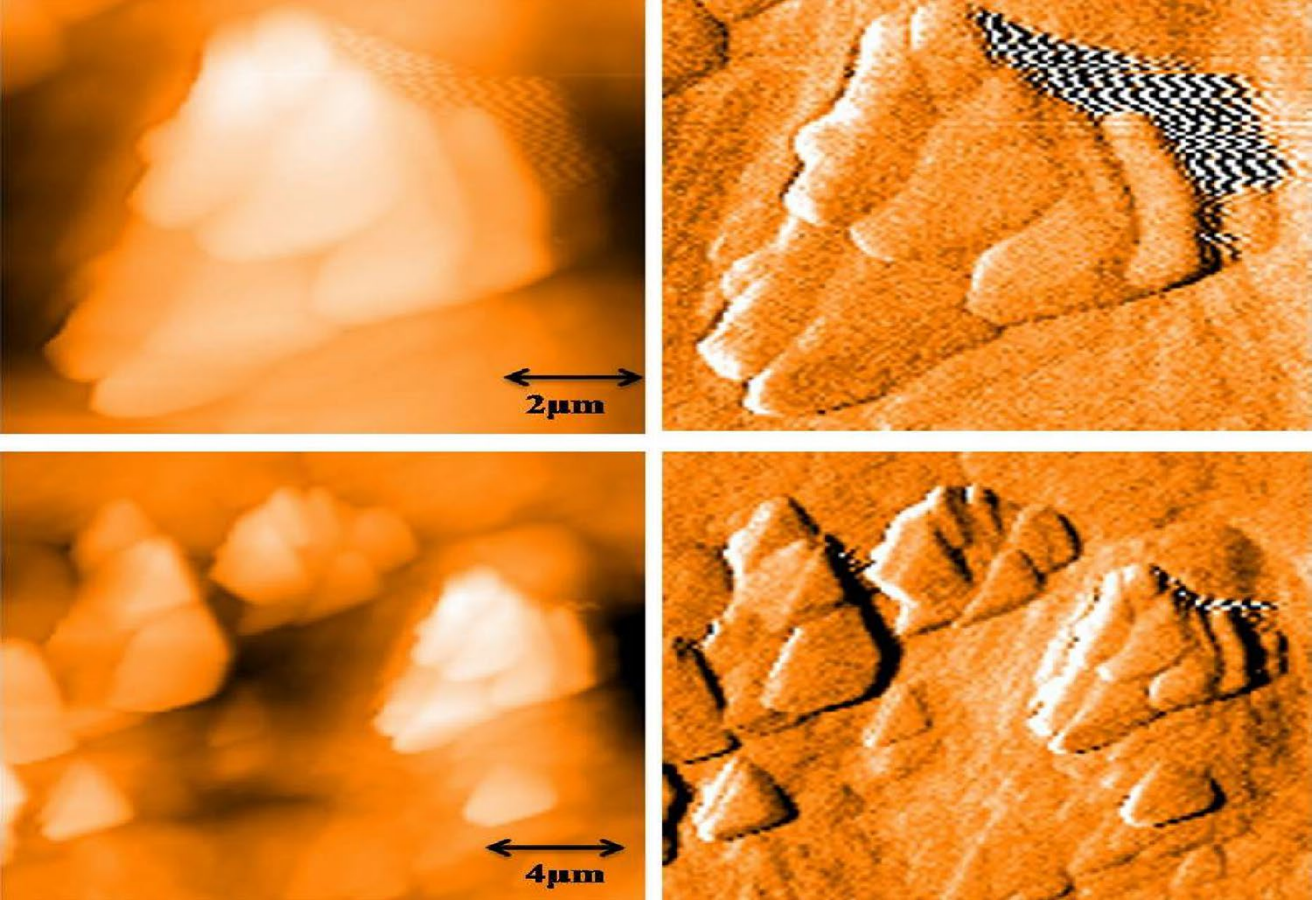
Atomic Force Microscope image of mangostan peel mediated synthesized gold nanoparticles at 2 µm and 4 µm.

Atomic force microscopy (AFM) was also known as scanning force microscopy (SFM). AFM is a basic technique and inevitable for all nanoscopic research. The AFM image of gold nanoparticles synthesized using the peel extract powder of mangostan is shown in Fig. 5. The particles synthesized agglomerated with each other to form a sheet-like form^39^. The particles were viewed at 2 and 4 µm distances and also in a 3D pattern. The particles synthesized using mangostan formed layers and are smaller.

#### Fourier transform infra-red spectroscopy

**Figure 6:**
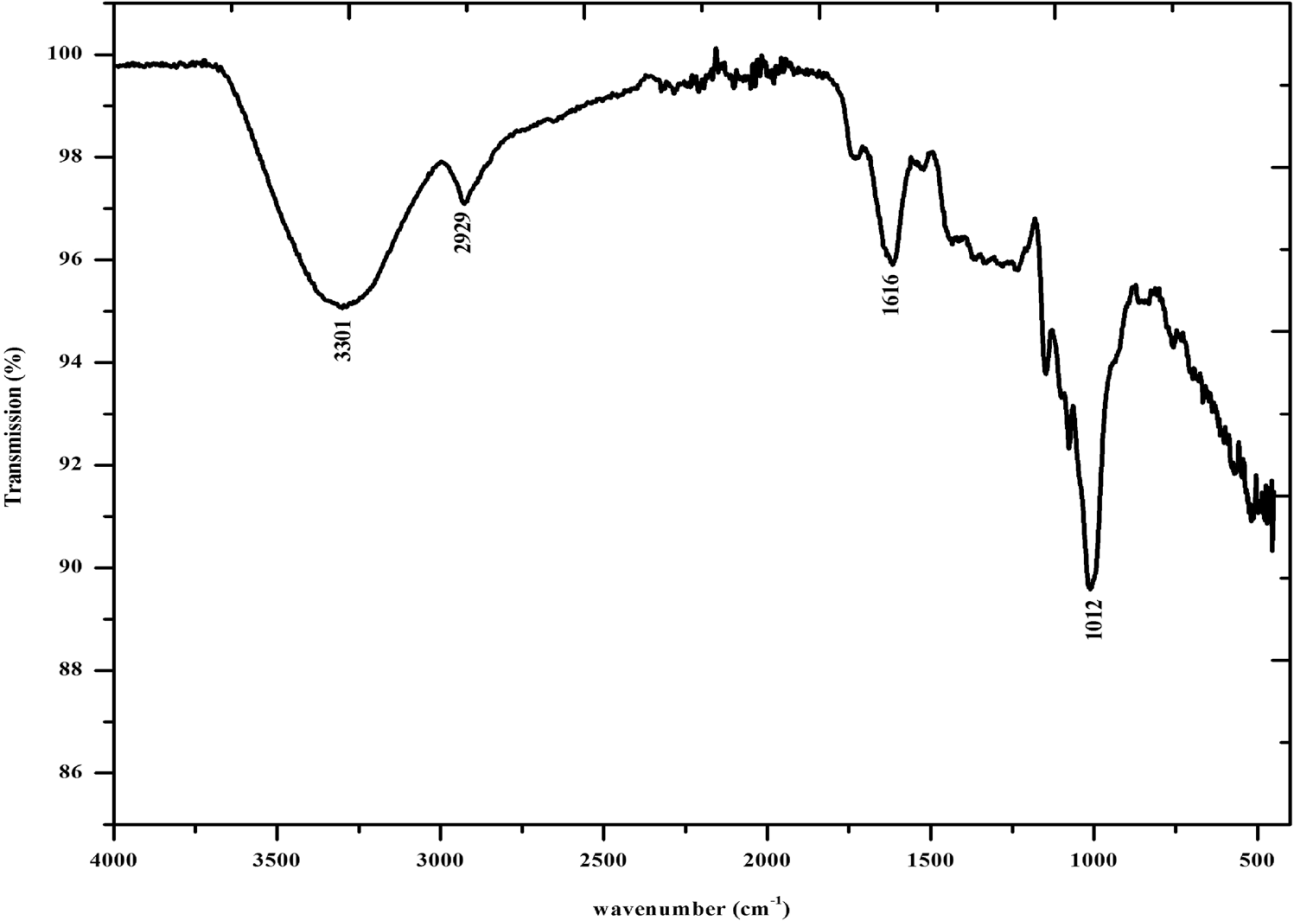
FTIR spectrum of gold nanoparticle.

The FTIR spectrum of gold nanoparticles synthesized using the peel extract powder of mangostan is presented in Fig. 6. The functional groups responsible for the synthesis of nanoparticles are denoted by the corresponding peaks arising due to the infrared radiations. The functional groups corresponding to the peak in the gold nanoparticles are presented in Table 1.

**Table 1:** Peak position and corresponding functional groups present in gold nanoparticles synthesized using mangostan peel extract powder.

S. no.	Peak position	Corresponding functional groups
1	3301	O–H stretch of alcohols, N–H stretch of 1°, 2° amines, amides
2	2929	O–H stretch of carboxylic acids, C–H stretch of alkanes
3	1616	N–H bend of 1° amines
4	1012	C–O stretch of carboxylic acids, alcohols, esters, ethers

#### Gas chromatography mass spectrum analysis

The GC–MS analysis revealed that eleven major constituents were present in the mangostan peel extract (Table 2). The compounds were first identified by Gas Chromatography and then the mass spectrum of the analyzed sample was matched with the NIST-2008 libraries. The analyses of mangostan peel extract indicate the presence of eleven major compounds with significant matches to 1, 3, 3 trimethyl, diaziridine (23.063%) followed by 2, 3 dimethyl, 1-pentanol (15.337%) and 11, 18-Diacetoxy-5, 6, 12, 17-Trinaphthylenetetrone (12.658%) major peak area. The compound names, molecular weight, empirical formula, retention time, peak area percentages, and structure of the identified compounds are given in Table 2.

**Table 2:** GCMS profile of compounds present in mangostan peel extract powder.

S. no.	Name	Molecular weight	Empirical formula	Retention time	Peak area %	Structure
1	1-(5-Hexenyl)-1-methyl hydrazine	128	C_7_H_16_N_2_	4.479	5.473	
2	11,18-Diacetoxy-5,6,12,17-Trinaphthylenetetrone	554	C_34_H_18_O_8_	4.754	12.658	
3	3-Amino-2-oxazolidinone	102	C_3_H_6_O_2_N_2_	4.939	5.543	
4	Mercaptamine	77	C_2_H_7_NS	5.054	5.544	
5	1-acetamido-2-nitro-guanidine	161	C_3_H_7_O_3_N_5_	5.304	4.736	
6	6-nitro 2-hexanol	147	C_6_H_13_O_3_N	5.539	7.260	
7	2,3 dimethyl, 1-pentanol	116	C_7_H_16_O	8.526	15.337	
8	1,3,3 trimethyl, diaziridine	86	C_4_H_10_N_2_	8.686	23.063	
9	5-methyl, 2-hexanol	116	C_7_H_16_O	8.846	10.062	
10	2-[(hexyloxy) methyl]- Oxirane	158	C_9_H_18_O_2_	8.961	10.323	
11	1-(3,5-Dimethyl-1-Adamantanoyl) Semi carbazide	265	C_14_H_23_O_2_N_3_	27.798	6.523	

### Dye fixing

#### Synthesis of various coloured gold nanoparticles

**Figure 7:**
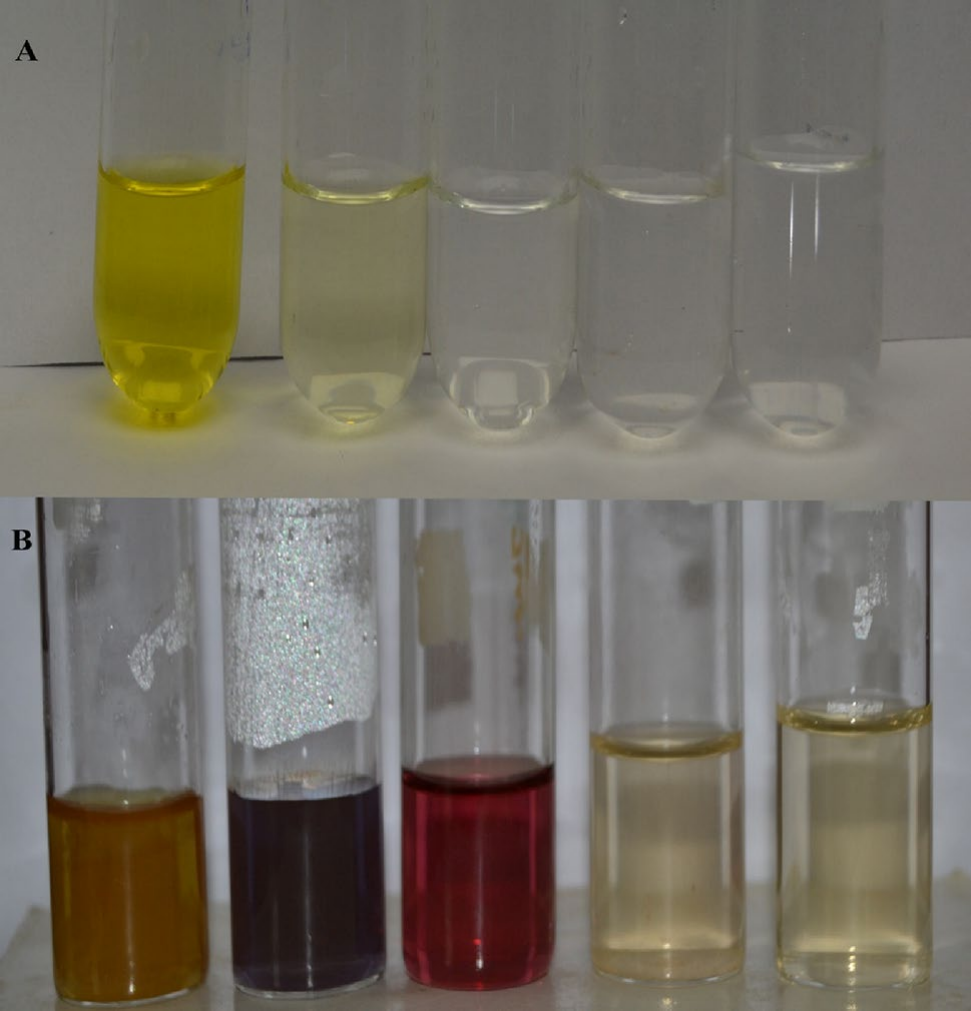
(**A**) Gold solution at various concentration, (**B**) gold nanoparticle colloid solutions.

The variety of attractive colors will be produced by altering the concentration of the gold ion solution as 10^–2^, 10^–3^, 10^–4^, 10^–5^, and 10^–6^ M solutions. They exhibit reddish-brown, purple, pink, pale yellow and colorless solutions respectively containing nanoparticles (Fig. 7). The particles exhibit different colors due to surface plasmon resonance effects in the visible region with the collective plasmon oscillations at the metal surface^40^. The rising of colors was based on the size of the gold nanoparticles. The colors of the cotton wicks were mainly various shades of brown, blue-purple, and shade blue colored fabrics produced.

#### Dyeing of cotton wicks

**Figure 8:**
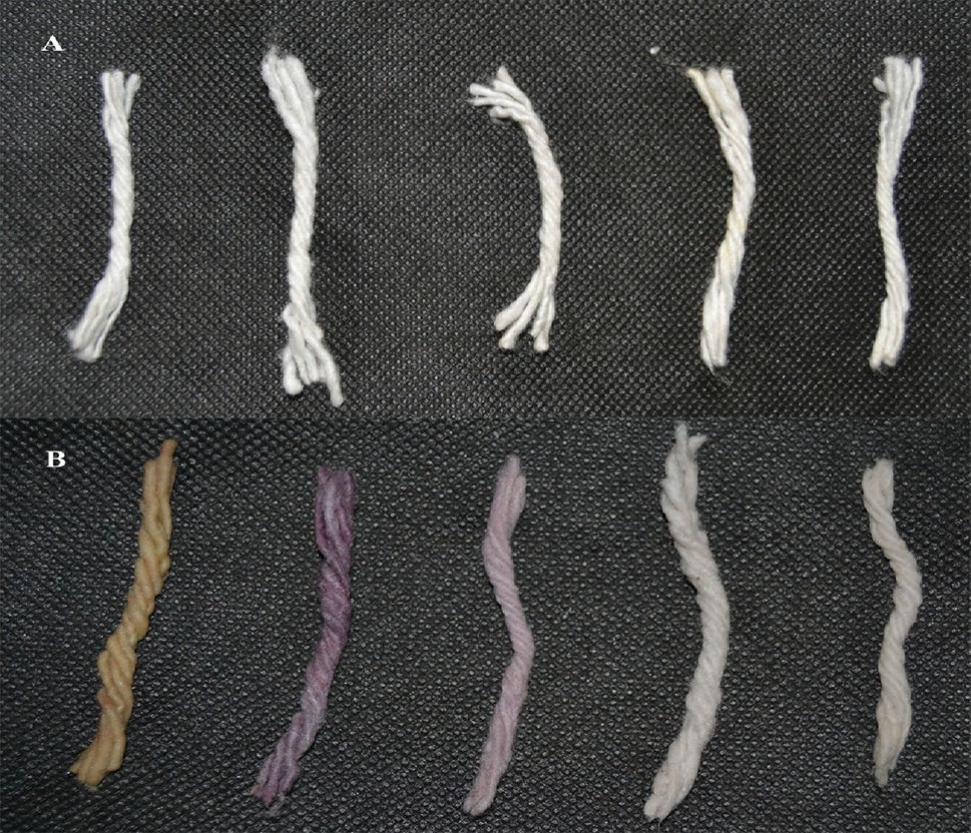
(**A**) cotton material before dyeing, (**B**) cotton material dyed using gold nanoparticle colloid solution.

The different colored gold nanoparticles can be readily attached to cotton wicks. Figure 8 shows the cotton wicks before and after the dyeing process. Purple and red-colored gold nanoparticle colloids were successfully attached with cotton wicks samples.

#### Colour fastness

**Figure 9:**
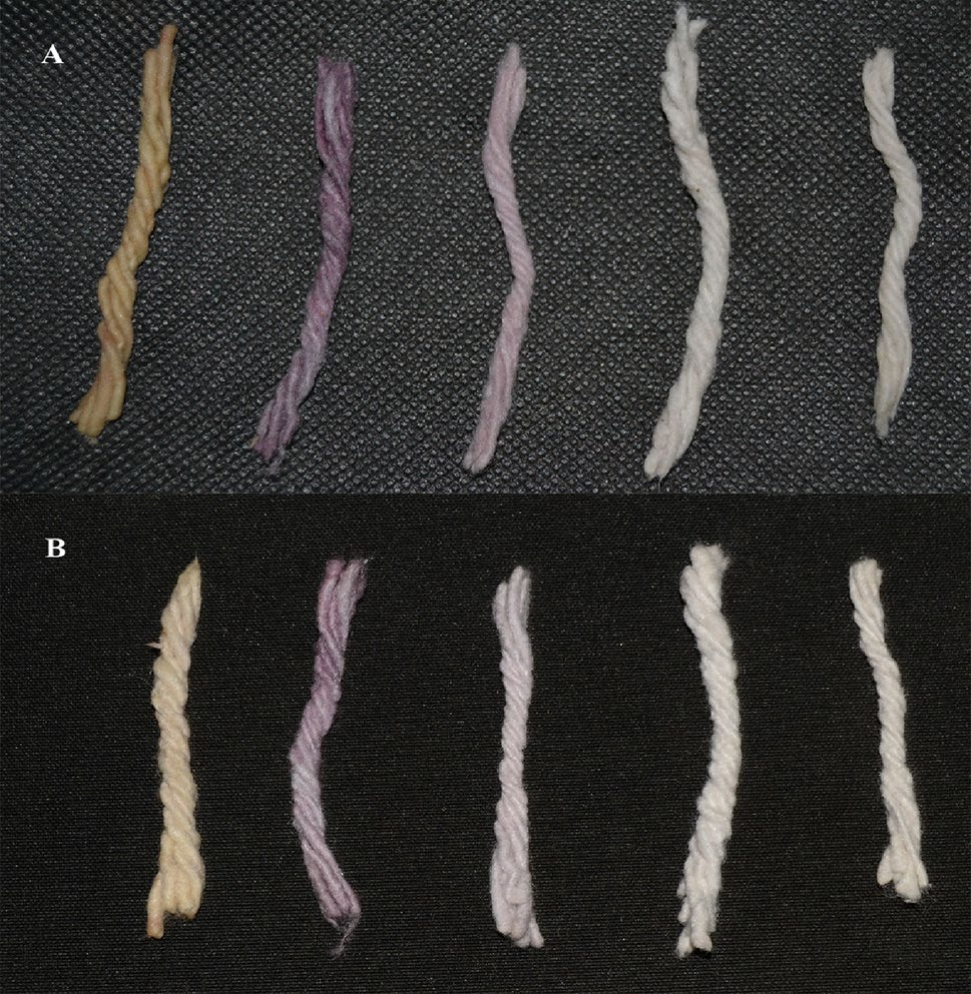
(**A**) cotton material dyed using gold nanoparticle colloid solution, (**B**) cotton material after exposure to various stress conditions.

Generally, some colors were degraded while exposed to UV light. So, the color fastness determines the quality of fibers^41^. Figure 9 shows the stable color of cotton wicks treated with gold nanoparticles after exposure to various conditions like acid, alkali, commercial detergent, and direct sunlight. The nanoparticles are embedded in the interspaces between the cross-linked fabric materials. This result indicates the dye is attached to fabrics and showed positive colorfastness because most dyes were still in a negative phase for colorfastness. Hence these results show that the cotton wicks are colorfast and stable so would be a viable alternative to current synthetic dyes.

#### Scanning electron microscope

**Figure 10:**
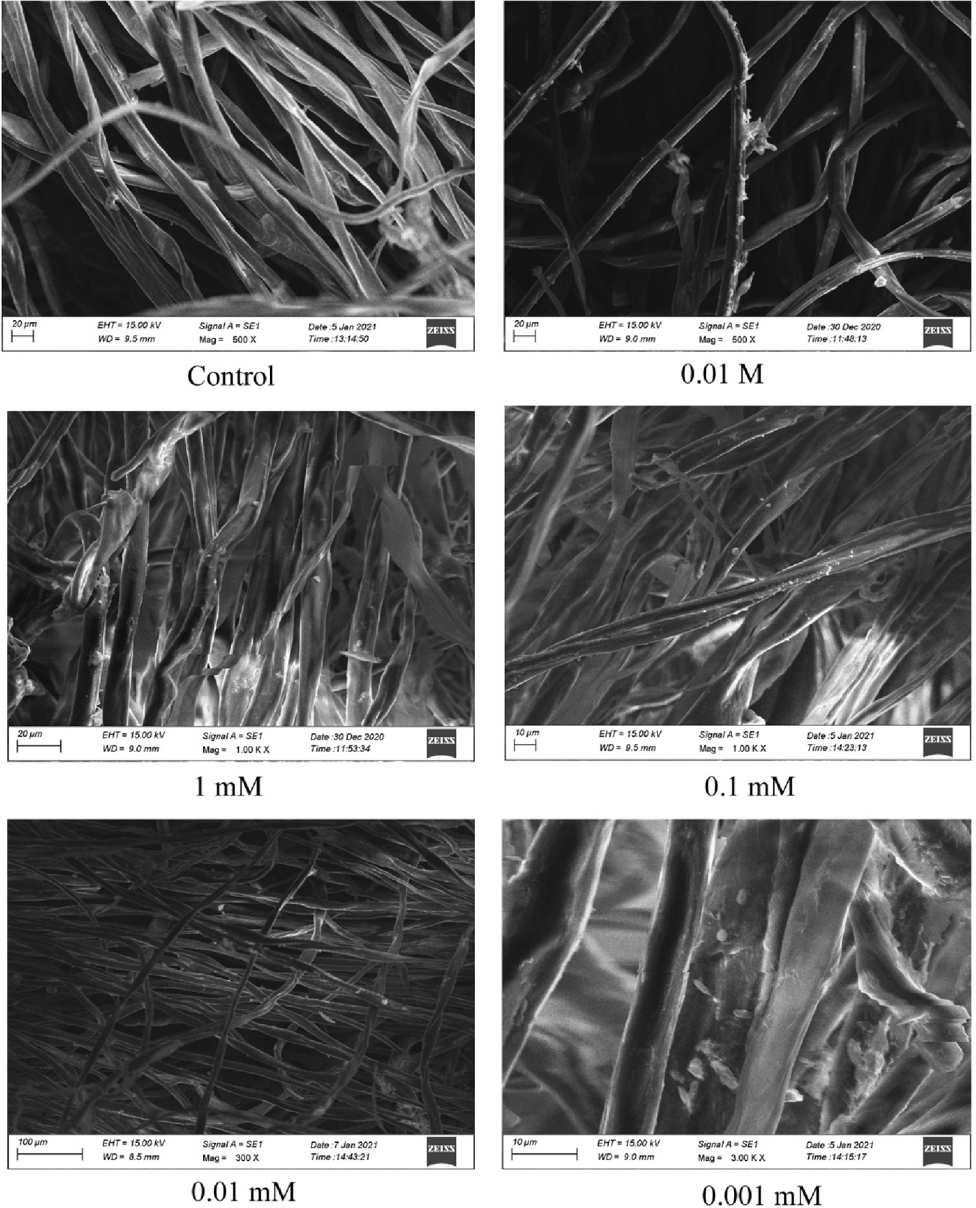
SEM image of gold nanoparticle dyed cotton material at various concentrations.

The SEM characterization technique is used to determine the morphology and physical properties of nanomaterials. The distribution of nanomaterials on the solid surface can also be attained using the SEM technique. SEM image (Fig. 10) visualizes the green synthesized gold nanoparticles which are deposited on the surface of cotton fabrics. Cotton fabrics are immobilized in gold nanoparticles prepared at different concentrations. Figure 10 shows the control and other concentrations used for coating the cotton fabrics with gold nanoparticle solution. The image depicts that many nanoparticles were adsorbed on the surface of the cotton fabrics with high aggregation. The particles are well dispersed and deposited on the surface of the cotton fabrics at 1 mM concentration of gold nanoparticles solution and other concentrations show the low distribution of gold nanoparticles. Moreover, gold nanoparticles were not identified on the surface of cotton fabrics treated with other low concentrations (0.1 mM, 0.01 mM, and 0.001 mM). The low concentration of gold nanoparticle solutions does not influence the binding of nanoparticles on the surface of cotton fabrics. As such, 1 mM gold nanoparticle solution was chosen as good results of our studies of immobilization of cotton fabrics.

## Conclusion

Gold nanoparticles impart different colors depending on their (Localized Surface Plasmon Resonance) LSPR in solution. The colors imparted by the nanoparticle solution were employed to dye cotton fabrics and the stability of the dyes on the fixed fabric was tested. These tests showed a positive result in the dyeing process. In summary, this paper presents an innovative approach to the synthesis and use of gold nanoparticles as novel stable colorants utilizing the surface plasmon resonance coloring effect of nanogold.
